# Machine Learning Models for Predicting Disability and Pain Following Lumbar Disc Herniation Surgery

**DOI:** 10.1001/jamanetworkopen.2023.55024

**Published:** 2024-02-07

**Authors:** Bjørnar Berg, Martin A. Gorosito, Olaf Fjeld, Hårek Haugerud, Kjersti Storheim, Tore K. Solberg, Margreth Grotle

**Affiliations:** 1 Centre for Intelligent Musculoskeletal Health, Faculty of Health Sciences, Oslo Metropolitan University, Oslo, Norway; 2 Division of Orthopedic Surgery, Oslo University Hospital, Oslo, Norway; 3 Department of Computer Science, Oslo Metropolitan University, Oslo, Norway; 4 Department of Neurology, Oslo University Hospital, Oslo, Norway; 5 Division of Clinical Neuroscience, Department of Research and Innovation, Oslo University Hospital, Oslo, Norway; 6 Institute of Clinical Medicine, The Artic University of Norway, Tromsø, Norway; 7 The Norwegian Registry for Spine Surgery, The University Hospital of North Norway, Tromsø, Norway

## Abstract

**Importance:**

Lumber disc herniation surgery can reduce pain and disability. However, a sizable minority of individuals experience minimal benefit, necessitating the development of accurate prediction models.

**Objective:**

To develop and validate prediction models for disability and pain 12 months after lumbar disc herniation surgery.

**Design, Setting, and Participants:**

A prospective, multicenter, registry-based prognostic study was conducted on a cohort of individuals undergoing lumbar disc herniation surgery from January 1, 2007, to May 31, 2021. Patients in the Norwegian Registry for Spine Surgery from all public and private hospitals in Norway performing spine surgery were included. Data analysis was performed from January to June 2023.

**Exposures:**

Microdiscectomy or open discectomy.

**Main Outcomes and Measures:**

Treatment success at 12 months, defined as improvement in Oswestry Disability Index (ODI) of 22 points or more; Numeric Rating Scale (NRS) back pain improvement of 2 or more points, and NRS leg pain improvement of 4 or more points. Machine learning models were trained for model development and internal-external cross-validation applied over geographic regions to validate the models. Model performance was assessed through discrimination (*C* statistic) and calibration (slope and intercept).

**Results:**

Analysis included 22 707 surgical cases (21 161 patients) (ODI model) (mean [SD] age, 47.0 [14.0] years; 12 952 [57.0%] males). Treatment nonsuccess was experienced by 33% (ODI), 27% (NRS back pain), and 31% (NRS leg pain) of the patients. In internal-external cross-validation, the selected machine learning models showed consistent discrimination and calibration across all 5 regions. The *C* statistic ranged from 0.81 to 0.84 (pooled random-effects meta-analysis estimate, 0.82; 95% CI, 0.81-0.84) for the ODI model. Calibration slopes (point estimates, 0.94-1.03; pooled estimate, 0.99; 95% CI, 0.93-1.06) and calibration intercepts (point estimates, −0.05 to 0.11; pooled estimate, 0.01; 95% CI, −0.07 to 0.10) were also consistent across regions. For NRS back pain, the *C* statistic ranged from 0.75 to 0.80 (pooled estimate, 0.77; 95% CI, 0.75-0.79); for NRS leg pain, the *C* statistic ranged from 0.74 to 0.77 (pooled estimate, 0.75; 95% CI, 0.74-0.76). Only minor heterogeneity was found in calibration slopes and intercepts.

**Conclusion:**

The findings of this study suggest that the models developed can inform patients and clinicians about individual prognosis and aid in surgical decision-making.

## Key Points

**Question:**

Can machine learning models accurately predict patient disability and pain following lumbar disc herniation surgery?

**Findings:**

In this prognostic study including 22 707 patients, machine learning models were developed and validated in large-scale, nationally representative data for treatment success or nonsuccess in disability and pain 12 months after lumbar disc herniation surgery. The models showed good discrimination and calibration.

**Meaning:**

The findings of this study suggest that algorithms can inform about individual prognosis and aid in surgical decision-making to ultimately reduce ineffective and costly spine care.

## Teaser

This prognostic study develops machine learning tools to use for prediction of patient pain and disability following lumbar disc herniation surgery.

## Introduction

The volume of lumbar spine surgery has increased considerably over the past decades, placing large costs on health care systems.^1,2,3^ Lumbar disc herniation surgery effectively reduces disability and pain in most patients, but a subset experience minimal benefit.^4,5,6^ In Scandinavia, 24% to 32% of patients do not achieve an important improvement in pain-related disability 1 year postoperatively.^7^ In most cases, the indication for surgery is relative. Therefore, shared decision-making, appraising both potential treatment risks and benefits, is essential to minimize ineffective and costly spine care.^8,9^ Providing precise probabilities of outcomes based on individual patient characteristics in a presurgical setting would allow clinicians to manage patients’ expectations before surgery and help patients make an informed choice about surgery.

Prediction models for disability and pain improvements following degenerative spine surgery have been developed; however, most studies including patients with lumbar disc herniation have limited generalizability due to a low number of patients from single surgical centers.^10,11,12,13,14,15^ Population-based spine registries with near complete national coverage hold a unique potential for prognostic modeling due to their comprehensive inclusion of a broad range of presurgical variables. Moreover, they reflect clinical practice settings and account for the uniqueness of a specific patient population.^16,17^

The volume and complexity of data available in national spine registries provide opportunities to develop better prediction models, which is necessary to improve quality of spine care.^16^ Machine learning algorithms are powerful tools for analyzing large amounts of data and have gained traction in recent years, but their use for outcome prediction in spine surgery remains nascent.^18^ The hope is that machine learning based on large and representative data can predict outcomes with high accuracy and consequently assist clinicians and patients in weighing the risks and benefits of surgical intervention. Therefore, the purpose of this study was to develop and validate machine learning models for predicting improvement in disability and pain 12 months after lumbar disc herniation surgery. We used internal-external cross-validation to evaluate generalizability over 4 geographic regions in Norway and a separate cluster for private hospitals.

## Methods

### Design

This was a multicenter study using prospectively collected data from adults undergoing surgery for lumbar disc herniation included in the Norwegian Registry for Spine Surgery (NORspine). We followed the methodologic framework proposed by the Prognosis Research Strategy group^19^ and report the study in line with the Transparent Reporting of a Multivariable Prediction Model for Individual Prognosis or Diagnosis (TRIPOD) reporting guideline.^20^ This study is part of the AID-Spine project, which has been approved by the ethics committee of the Health Region of South-East Norway. Written informed consent was obtained from all patients in the NORspine, and the Data Protection Authority of Norway approved the registry protocol.

### Data Source and Patient Population

NORspine is a comprehensive clinical registry for degenerative spine surgery designed for quality control and research. The register is mandatory and had a coverage of 100% at the surgical unity level in 2021 (40 centers). The individual-level completeness rate was 81% for lumbar spine surgery in 2021.^21^ All patients included in the NORspine registry who had undergone lumbar discectomy from January 1, 2007, to May 31, 2021, were identified and screened for eligibility. Repeat surgeries were included as new cases if performed more than 90 days after the index surgery. Reoperation within 90 days is considered a complication in the NORspine registry, and thus not recorded as a new case. The registry also excludes patients who undergo an operation for fractures, trauma, or cancer.^21^ Patients with cauda equina syndrome were excluded from the current study.

The NORspine data collection process comprised a preoperative form including patient-reported outcomes to be completed by the patients at the time of surgical admission (baseline). Information regarding diagnosis, previous lumbar spine surgery, comorbidity, imaging findings, and surgical procedure were recorded by the surgeon on a standardized form. At 12 months postoperatively, follow-up questionnaires including patient-reported outcomes were distributed by mail to the patients.

### Outcomes

The outcomes included measured patient improvements using the Oswestry Disability Index (ODI)^22^ and Numeric Rating Scale (NRS)^23^ for back pain and leg pain from baseline to 12 months. The ODI was the primary outcome; it is a 10-item score from 0 (none) to 100 (maximum disability) encompassing limitations in various activities of daily living.^22^ The NRS measures pain intensity during the last week on an 11-point scale, with 0 representing no pain and 10 the worst imaginable pain.^23^

The outcomes were operationalized as treatment success, with study-specific calculations of the cutoffs for success. The thresholds were arrived at using the anchor-based predictive modeling method,^24^ adjusted for the proportion of patients reporting improvement.^25^ As the dichotomized anchor response, we used a 7-point Global Perceived Effect scale,^26^ with the cutoff for success and nonsuccess set between patients responding they were much improved vs slightly improved. The cutoff scores were: ODI, 22 points; NRS back pain, 2 points; and NRS leg pain, 4 points improvement from baseline to 12 months.

### Predictors

We used all potential preoperative predictors available in the NORspine registry (eTable 1 in Supplement 1 provides a detailed description). In short, 25 features (predictor variables) were included, covering patient demographic characteristics, comorbidity, clinical characteristics, analgesics use, and type of operation. Only preoperative features were included, given our aim of improving the selection of surgical candidates.

### Sample Size

Our sample size was restricted to available NORspine data. An a priori sample size calculation was performed to evaluate the adequacy of the data set and guide how many predictors could be included.^27^ We assumed an event rate of 30% (nonsuccess at 12 months),^7^ a *C* statistic of 0.75 based on a recent systematic review,^28^ and a maximum number of 50 predictor parameters. Based on these inputs, a sample size of 2551 patients would be required for the model development, corresponding to 766 events and an event per parameter of 15.3. The pmsampsize package in Stata, version 17.1 (StataCorp LLC) was used for the calculations,^27^ with Cox-Snell *R*^2^ value estimated from the *C* statistic.^29^ While the number of predictor parameters for machine learning likely exceeds that for regression, our sample size far exceeds the minimum requirement estimate for regression-based prediction models.^27^

### Data Cleaning and Quality Checks

The NORspine registry data quality is periodically assessed by the registry owner to detect systematic or random errors in the data entry.^30^ We performed further data quality checks during data cleaning, including assessment of potential duplicate entries, outliers, the extent of missing data, and presence of systematically missing variables within and across clusters. All patient characteristics and model predictors were determined at the time of surgical admission. The same eligibility criteria and characteristic determination methods were applied to all clusters.

### Statistical Analysis

Data analysis was performed from January to June 2023. We calculated descriptive statistics for baseline characteristics, overall and for each cluster separately. We applied multiple imputation with chained equations to handle missing baseline and outcome data, which were assumed to be missing at random, with 50 imputed data sets generated. The imputation models included all features and outcomes, performed separately for each cluster to allow the distribution of the imputed values to differ among clusters.^31^ Imputations were assessed for consistency by comparing distributions of imputed values with the complete data. The predictive performance measures were estimated in each imputed data set separately before being combined across imputations using the Rubin rule.^32^

Seven supervised machine learning algorithms were trained to develop the prediction model: random forest, logistic regression, linear discriminant analysis, multilayer perceptron, gradient boosting, extra trees, and extreme gradient boosting. Preprocessing steps involved scaling of continuous variables (minimum-maximum normalization) and 1-hot encoding of categorical variables. All features were included, ie, no variable selection techniques were used. Continuous variables were kept continuous to avoid loss of prognostic information. Hyperparameters were tuned using a grid search with 5-fold cross-validation (eTable 2 in Supplement 1). The best algorithm for each outcome was estimated based on model discrimination using the *C* statistic. We assessed apparent performance (using the same data in which the model was developed), quantified with the *C* statistic.

Internal-external cross-validation was used to evaluate the derived prediction models to give a more realistic estimate of model performance and heterogeneity in performance across regions.^33,34^ Internal-external cross-validation involves a nonrandom split of data based on clusters, in our case, 4 geographic regions corresponding to the 4 Norwegian Regional Health Authorities and an additional cluster for private hospitals. A single internal-external cross-validation cycle separates the data into a development cohort and validation cohort, with 4 of 5 clusters forming the development cohort and reserving the other cluster for validation. The process was repeated 5 times, each time reserving a different cluster for validation.

We calculated *C* statistics, positive predictive value, negative predictive value, calibration slopes, and calibration intercepts in each cluster. Overall performance measures with 95% CIs (derived using the Hartung-Knapp-Sidik-Jonkman variance correction) and 95% prediction intervals were also summarized across clusters using a random-effects meta-analysis.^35,36^ We further present calibration plots with comparison of observed to predicted risk for each model overall and by validation cluster (created using pmcalplot on Stata), generated separately in each imputed data set and checked for consistency across imputations. Clinical utility was examined using decision curve analysis by comparing the prediction models against blanket treatment strategies to treat all or to treat none at varying risk thresholds.^37^ Shapley Additive Explanations values were calculated for all 3 prediction models to investigate feature importance.^38^ The machine learning algorithms were implemented using Python 3.8.13 and Scikit-learn Python libraries. Stata was used for data cleaning and multiple imputation. As a sensitivity analysis, results obtained from using imputed data were compared with those of complete case analysis for each outcome.

## Results

**Figure 1.**
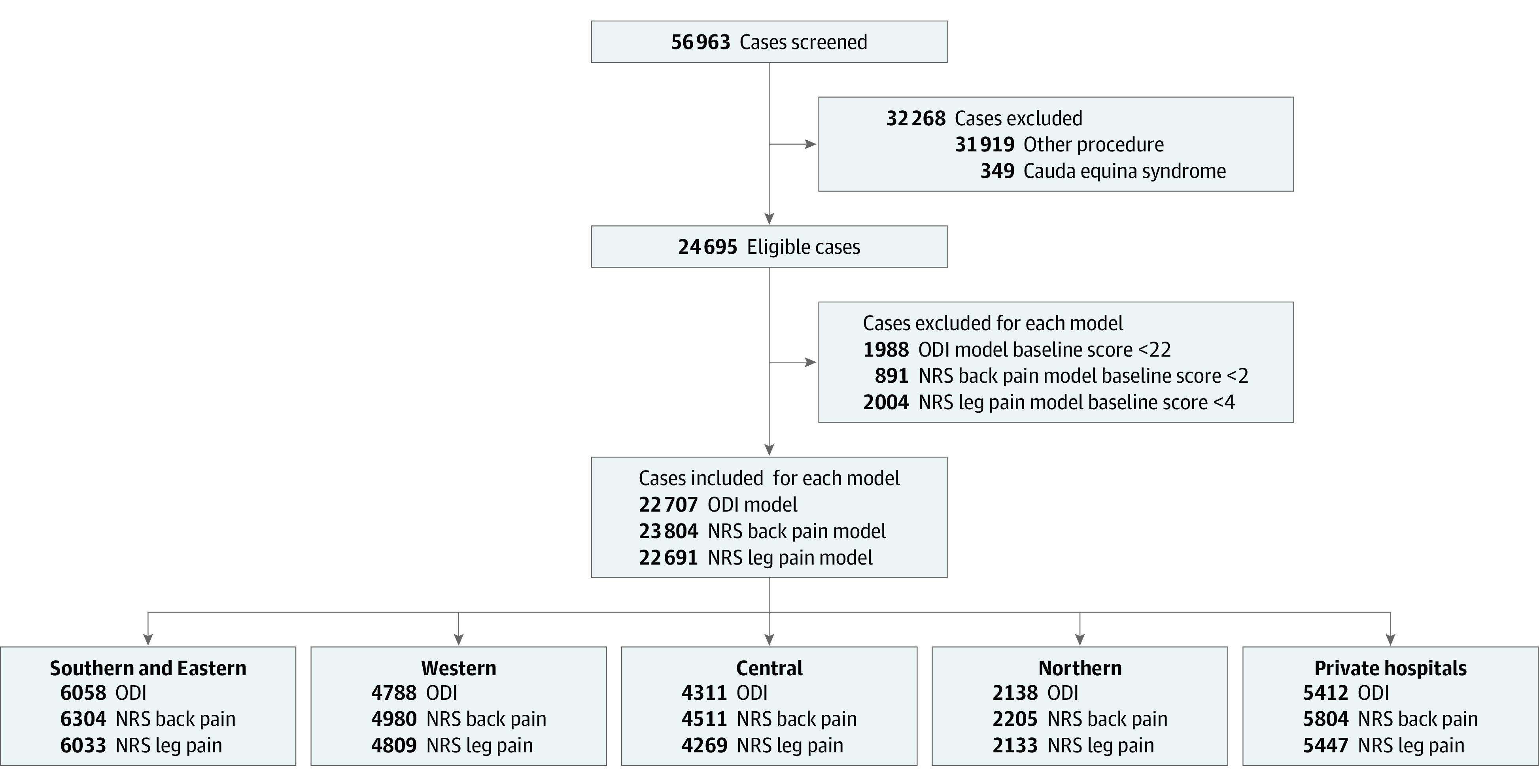
Flow Diagram of Surgical Cases Included in the Analysis NRS indicates Numeric Rating Scale; ODI, Oswestry Disability Index.

Of 56 963 surgical cases screened, we identified 22 707 surgical cases (21 161 patients) (mean [SD] age, 47.0 [14.0] years; 12 952 [57.0%] males; 9755 females [43.0%]) who underwent operations for lumbar disc herniation eligible for inclusion in our primary analysis (Figure 1). Baseline characteristics of the total study population and stratified by cluster for the ODI model are summarized in the Table. The analysis for NRS back pain included 23 804 cases and, for NRS leg pain, 22 691 cases. The proportions of cases experiencing treatment nonsuccess were 33% (ODI), 27% (NRS back pain), and 31% (NRS leg pain).

**Table.** Study Population for the ODI Model

Characteristic	No. (%)					
	Development, total (n = 22 707)	Cluster				
		Southern and Eastern (n = 6058)	Western (n = 4788)	Central (n = 4311)	Northern (n = 2138)	Private hospitals (n = 5412)
ODI^a^						
Treatment success	9934 (66.9)	2363 (60.4)	2032 (65.8)	1966 (67.3)	1086 (72.6)	2487 (72.3)
Missing	7848 (34.6)	2145 (35.4)	1699 (35.5)	1388 (32.2)	643 (30.1)	1973 (36.5)
Sex						
Male	12 952 (57.0)	3139 (51.8)	2540 (53.1)	2339 (54.3)	1247 (58.3)	3687 (68.1)
Female	9755 (43.0)	2919 (48.2)	2248 (46.9)	1972 (45.7)	891 (41.7)	1725 (31.9)
Age, mean (SD), y	47.0 (14.0)	47.3 (14.3)	47.9 (14.9)	48.3 (14.7)	45.3 (13.2)	45.6 (12.2)
BMI, mean (SD)	27.0 (4.5)	27.2 (4.7)	26.9 (4.5)	27.2 (4.5)	27.3 (4.7)	26.7 (4.0)
Missing	1327 (5.8)	253 (4.2)	113 (2.4)	500 (11.6)	84 (3.9)	377 (7.0)
Nonnative language speaker	1617 (7.2)	541 (9.0)	393 (8.3)	221 (5.2)	120 (5.6)	342 (6.4)
Missing	129 (0.6)	45 (0.7)	29 (0.6)	28 (0.7)	4 (0.2)	23 (0.4)
Marital status, single	5583 (24.8)	1670 (27.9)	1216 (25.6)	1017 (23.9)	539 (25.4)	1141 (21.2)
Missing	205 (0.9)	71 (1.2)	33 (0.7)	55 (1.3)	17 (0.8)	29 (0.5)
Smoker	5797 (25.8)	1604 (26.8)	1273 (26.8)	1134 (26.6)	606 (28.7)	1180 (22.0)
Missing	216 (1.0)	71 (1.2)	33 (0.7)	55 (1.3)	17 (0.8)	29 (0.5)
Education						
Lower secondary school	3255 (14.5)	902 (15.1)	790 (16.7)	692 (16.3)	375 (17.7)	496 (9.2)
Upper secondary school	10 575 (47.1)	2793 (46.7)	2237 (49.3)	2072 (48.8)	991 (46.8)	2392 (44.5)
University (1-3 y)	4606 (20.5)	1192 (20.0)	855 (18.1)	791 (18.6)	385 (18.2)	1383 (25.7)
University (≥4 y)	4003 (17.8)	1089 (18.2)	747 (15.8)	692 (16.3)	368 (17.4)	1107 (20.6)
Missing	268 (1.2)	82 (1.4)	69 (1.4)	64 (1.5)	19 (0.9)	34 (0.6)
Work status						
Working/student	5943 (26.8)	1498 (25.4)	1168 (24.8)	972 (23.2)	514 (24.7)	1791 (34.0)
Retirement age^b^	2277 (10.3)	676 (11.5)	618 (13.1)	545 (13.0)	156 (7.5)	282 (5.4)
Sick leave	10 871 (49.1)	2717 (46.1)	2165 (45.9)	1972 (47.1)	1103 (53.0)	2914 (55.4)
Disability pension	3058 (13.8)	1005 (17.1)	767 (16.3)	702 (16.8)	309 (14.8)	275 (5.2)
Missing	558 (2.5)	162 (2.7)	70 (1.5)	120 (2.8)	56 (2.6)	150 (2.8)
Litigation issue^c^	1723 (7.6)	508 (8.4)	320 (6.7)	324 (7.5)	168 (7.9)	403 (7.5)
Anxiety or depression^d^	8844 (39.8)	2444 (41.4)	1942 (41.3)	1801 (42.7)	873 (41.5)	1784 (33.7)
Missing	490 (2.2)	158 (2.6)	90 (1.9)	91 (2.1)	34 (1.6)	117 (2.2)
Comorbidities						
0	16 922 (74.5)	4366 (72.1)	3277 (68.4)	3098 (71.9)	1709 (79.9)	4472 (82.6)
1	3879 (17.1)	1153 (19.0)	922 (19.3)	805 (18.7)	296 (13.8)	703 (13.0)
2	1363 (6.0)	379 (6.3)	406 (8.5)	291 (6.8)	100 (4.7)	187 (3.5)
≥3	543 (2.4)	160 (2.6)	183 (3.8)	117 (2.7)	33 (1.5)	50 (0.9)
ASA grade ≥3	1489 (6.6)	456 (7.6)	359 (7.6)	441 (10.4)	90 (4.4)	143 (2.7)
Missing	297 (1.3)	49 (0.8)	34 (0.7)	82 (1.9)	71 (3.3)	61 (1.1)
ODI (0-100), mean (SD)	48.6 (17.2)	48.2 (16.8)	50.9 (17.5)	50.3 (17.8)	52.5 (18.7)	44.3 (15.2)
Missing	178 (0.8)	57 (0.9)	34 (0.7)	23 (0.5)	8 (0.4)	56 (1.0)
NRS pain intensity^e^						
Back pain	6.6 (2.4)	6.5 (2.30	6.8 (2.3)	6.7 (2.3)	6.9 (2.4)	6.1 (2.3)
Missing	682 (3.0)	217 (3.6)	156 (3.3)	120 (2.8)	46 (2.2)	143 (2.6)
Leg pain	7.2 (2.1)	7.1 (2.1)	7.4 (2.0)	7.3 (2.1)	7.5 (2.1)	6.9 (2.0)
Missing	666 (2.9)	206 (3.4)	157 (3.3)	130 (3.0)	40 (1.9)	133 (2.5)
EQ-5D, mean (SD)	0.48 (0.22)	0.49 (0.22	0.45 (0.22)	0.47 (0.22)	0.44 (0.23)	0.53 (0.19)
Missing	950 (4.2)	307 (5.1)	191 (4.0)	197 (4.6)	62 (2.9)	193 (3.6)
EQ VAS (0-100), mean (SD)	43.2 (21.1)	44.1 (21.1)	40.6 (21.0)	42.2 (21.5)	41.8 (22.1)	45.9 (20.1)
Missing	1278 (5.6)	396 (6.5)	306 (6.4)	285 (6.6)	83 (3.9)	208 (3.8)
Back pain, mo						
<3	4196 (19.2)	765 (13.1)	928 (19.9)	848 (20.7)	608 (29.3)	1047 (20.2)
3-11	9797 (44.8)	2625 (45.1)	2046 (43.8)	1730 (42.1)	800 (38.5)	2596 (50.0)
12-24	3166 (14.5)	996 (17.1)	678 (14.5)	612 (14.9)	271 (13.0)	609 (11.7)
>24	4715 (21.6)	1441 (24.7)	1015 (21.8)	917 (22.3)	399 (19.2)	943 (18.2)
Missing	833 (3.7)	231 (3.8)	121 (2.5)	204 (4.7)	60 (2.8)	217 (4.0)
Leg pain, mo						
<3	5547 (25.6)	1055 (18.3)	1258 (27.3)	1101 (27.1)	773 (37.6)	1360 (26.3)
3-11	10 813 (49.9)	2962 (51.5)	2232 (48.4)	1915 (47.1)	847 (41.2)	2857 (55.2)
12-24	2760 (12.7)	917 (15.9)	597 (12.9)	524 (12.9)	228 (11.1)	494 (9.5)
>24	2548 (11.8)	823 (14.3)	527 (11.4)	524 (12.9)	209 (10.2)	465 (9.0)
Missing	1039 (4.6)	301 (5.0)	174 (3.6)	247 (5.7)	81 (3.8)	236 (4.4)
Analgesic use						
Monthly	3964 (17.8)	1044 (17.6)	728 (15.4)	726 (17.2)	322 (15.2)	1144 (21.5)
Weekly	2883 (12.9)	770 (13.0)	516 (10.9)	496 (11.7)	234 (11.0)	867 (16.3)
Daily	15 471 (69.3)	4122 (69.4)	3478 (73.7)	3007 (71.1)	1566 (73.8)	3301 (62.1)
Missing	389 (1.7)	122 (2.0)	66 (1.4)	82 (1.9)	19 (0.9)	100 (1.9)
Paresis grade						
Normal	18 469 (81.3)	5217 (86.2)	3867 (80.8)	3635 (84.3)	1480 (69.2)	4270 (78.9)
Mild	2683 (11.8)	504 (8.3)	593 (12.4)	378 (8.8)	433 (20.3)	775 (14.3)
Severe	1555 (6.9)	337 (5.6)	328 (6.9)	298 (6.9)	225 (10.5)	367 (6.8)
Previous surgery						
0	17 469 (77.5)	4765 (79.2)	3626 (75.9)	3135 (73.5)	1704 (80.4)	4239 (79.0)
1	3924 (17.4)	991 (16.5)	863 (18.1)	803 (18.8)	334 (15.8)	933 (17.4)
≥2	1160 (5.1)	261 (4.3)	291 (6.1)	330 (7.7)	82 (3.9)	196 (3.7)
Missing	154 (0.7)	41 (0.7)	8 (0.2)	43 (1.0)	18 (0.8)	44 (0.8)
Microdiscectomy	21 255 (93.6)	5523 (91.2)	4338 (90.6)	4065 (94.3)	2114 (98.9)	5215 (96.4)
Surgical levels ≥2	1263 (5.6)	466 (7.7)	246 (5.1)	124 (2.9)	91 (4.3)	336 (6.2)
Emergency surgery	4397 (19.5)	1022 (17.1)	1366 (28.6)	1018 (23.7)	921 (43.3)	70 (1.3)
Missing	131 (0.6)	68 (1.1)	12 (0.3)	16 (0.4)	10 (0.5)	25 (0.5)

Abbreviations: ASA, American Society of Anesthesiologists; BMI, body mass index (calculated as weight in kilograms divided by height in meters squared); NRS, Numeric Rating Scale; ODI, Oswestry Disability Index.

^a^ A 10-item score from 0 (none) to 100 (maximum disability) encompassing limitations in various activities of daily living. Treatment success is defined based on achievement of the minimal important change (≥22 points improvement from baseline).

^b^ Individuals receiving retirement/age pension. While the retirement age in Norway is 67 years, individuals have the flexibility to decide when they wish to start receiving their retirement pension.

^c^ Pending medical or insurance claim or litigation issue.

^d^ EQ-5D questionnaire; 5th item, moderate to severe (3L) or moderate to extreme (5L).

^e^ The NRS measures pain intensity during the last week on an 11-point scale, with 0 representing no pain and 10 the worst imaginable pain

No features had more than 6% missing values. The proportions with missing outcome data were 35% (ODI) and 37% (NRS back pain and NRS leg pain).

### Model Development

The predictive performance of the 7 different machine learning algorithms was compared using estimates of the random effects meta-analysis per algorithm and outcome. The difference between the maximum and minimum *C* statistic was only 0.01 for each outcome. However, calibration intercepts and slopes varied substantially across the algorithms (eTable 3 in Supplement 1). Extreme gradient boosting had the highest discriminatory performance for each outcome while also showing excellent calibration.

### Apparent Predictive Model Performance

The ODI model was able to discriminate between patients with treatment success and nonsuccess with an apparent *C* statistic of 0.83 (95% CI, 0.82-0.84). The *C* statistic was 0.78 (95% CI, 0.77-0.78) for NRS back pain and 0.76 (95% CI, 0.76-0.77) for NRS leg pain.

### Internal-External Cross-Validation

**Figure 2.**
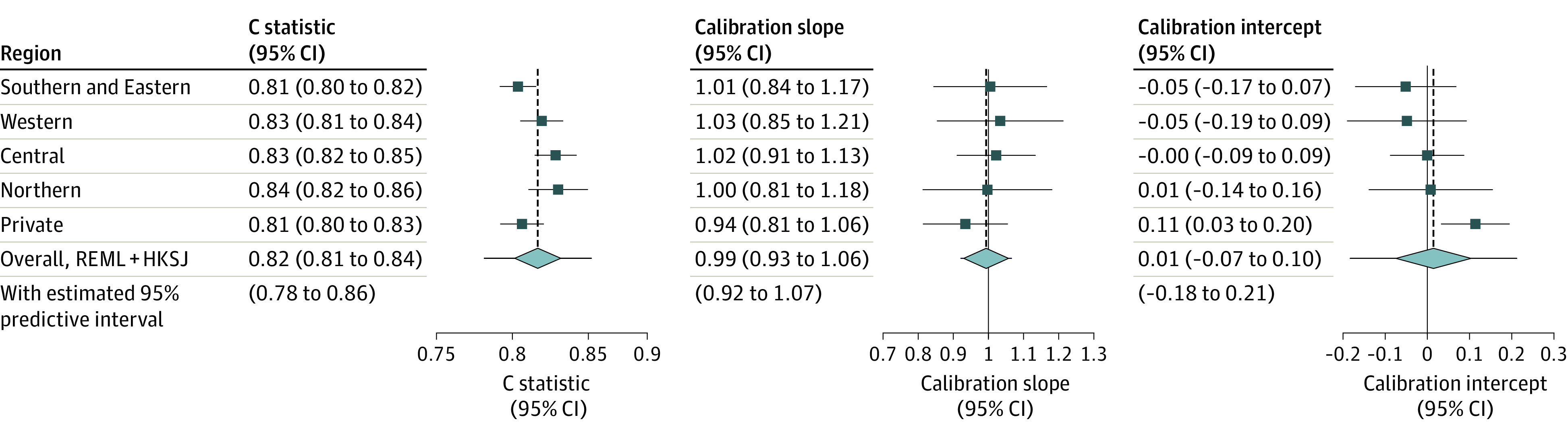
Internal-External Cross-Validation in 5 Validation Cohorts and the Overall Estimation Across Validation Cohorts for Oswestry Disability Index REML+ HKSJ indicates restricted maximum likelihood + Hartung-Knapp-Sidik-Jonkman.

**Figure 3.**
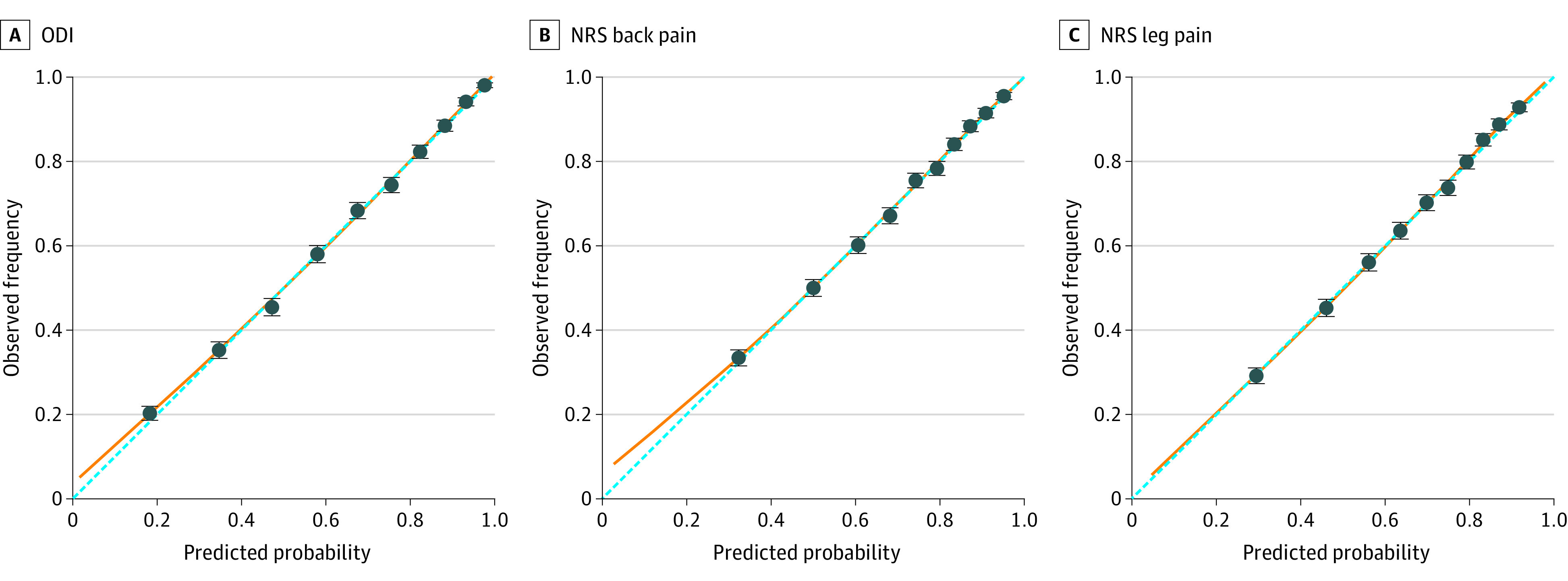
Assessment of Overall Calibration The dashed blue line indicates perfect calibration. The orange line is a fitted Loess smoother curve for the predicted probabilities. NRS indicates Numeric Rating Scale; ODI, Oswestry Disability Index.

Model discrimination (*C* statistic) and calibration metrics (slope and intercept) from internal-external cross-validation of the ODI model are shown in Figure 2. *C* statistics were similar across regions, with point estimates ranging from 0.81 to 0.84 (pooled random-effects meta-analysis estimate, 0.82; 95% CI, 0.81-0.84). Positive predictive values ranged from 0.81 to 0.88 (pooled estimate, 0.86; 95% CI, 0.82-0.89) and negative predictive values ranged from 0.51 to 0.63 (pooled estimated, 0.58; 95% CI, 0.52-0.64) (eTable 4 in Supplement 1). Calibration slopes were consistent across regions (point estimates, 0.94-1.03; pooled estimate, 0.99; 95% CI, 0.93-1.06). There was minor heterogeneity in calibration intercept across regions, likely due to some variation in outcome incidence between regions (point estimates, −0.05 to 0.11; pooled estimate, 0.01; 95% CI, −0.07 to 0.10). The overall calibration plot for the ODI model is shown in Figure 3A and by region in eFigure 2A in Supplement 1.

For NRS back pain and NRS leg pain, discrimination was somewhat lower, with *C* statistics ranging from 0.75 to 0.80 (pooled estimate, 0.77; 95% CI, 0.75-0.79) for NRS back pain and 0.74 to 0.77 (pooled estimate, 0.75; 95% CI, 0.74-0.76) for NRS leg pain (eFigure 1 in Supplement 1). Predictive values are reported in eTable 4 in Supplement 1. The calibration slope was also similar across regions, ranging from 0.96 to 1.09 for NRS back pain and 0.91 to 1.10 for NRS leg pain. After meta-analysis, the summary calibration slope was 1.01 (95% CI, 0.94-1.07) for NRS back pain and 1.02 (95% CI, 0.92-1.12) for NRS leg pain. Calibration intercept was consistent across regions for NRS back pain (point estimate, −0.06 to 0.08; pooled estimate, 0.00; 95% CI, −0.07 to 0.08). For NRS leg pain (point estimate, −0.09 to 0.14; pooled estimate, −0.01; 95% CI, −0.14 to 0.11), the overall risk was underestimated in private hospitals (0.14; 95% CI, 0.03-0.25). The NRS back pain and NRS leg pain overall calibration plots are shown in Figure 3B and C and by region in eFigure 2B and C in Supplement 1.

Decision curve analyses in the validation sets from internal-external validation are shown in eFigure 3 in Supplement 1. For all outcomes, the prediction model had higher net benefit than the treat-all or treat-none strategies across a broad range of threshold probabilities.

### Feature Importance

**Figure 4.**
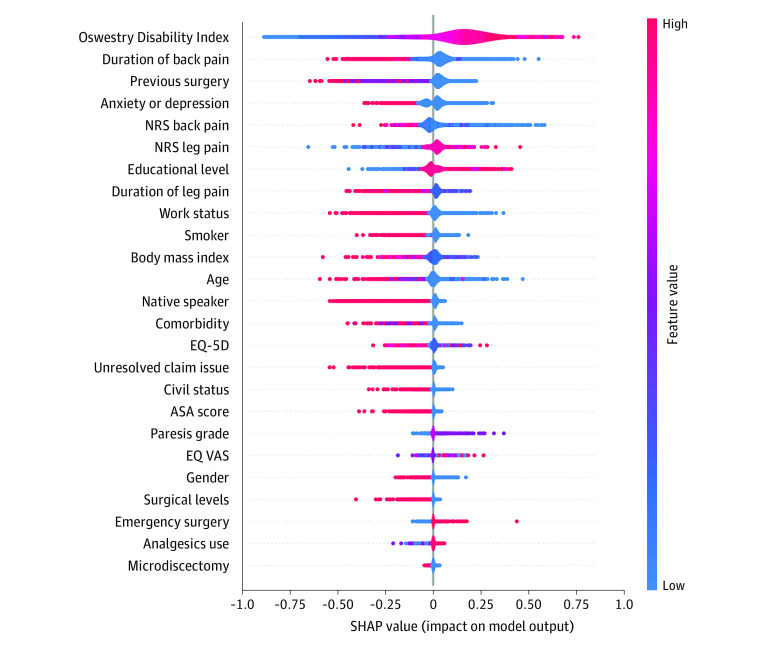
Shapley Additive Explanations (SHAP) Summary Plot of Variable Importance for the Oswestry Disability Index Model Predictive features are arranged based on their importance. Each dot represents 1 prediction result. SHAP values indicate the distribution of the prediction among the features; a positive value contributes to treatment success, while a negative value contributes to nonsuccess. ASA indicates American Society of Anesthesiologists; and NRS, Numeric Rating Scale.

The most important features for treatment success on the ODI were higher baseline score of the outcome, shorter duration of back pain, no previous surgery, and no symptoms of anxiety and depression (Figure 4). The same features were also among the most influential for NRS back pain and NRS leg pain (eFigure 4 and eFigure 5 in Supplement 1).

### Sensitivity Analyses

Consistent results were found for all 3 models in sensitivity analyses including only surgical cases with complete data (n = 11 461 for ODI, n = 11 944 for NRS back pain, and n = 11 321 for NRS leg pain). Calibration slopes, calibration intercept, and *C* statistics are shown in eFigure 6 in Supplement 1. (eFigure 6 in Supplement 1).

## Discussion

In this prognostic study, we developed and validated machine learning models for prediction of treatment success or nonsuccess 12 months after lumbar discectomy. Internal-external cross-validation showed that our models had consistently good calibration when applied to the different geographic regions and private hospitals, and good discrimination with *C* statistics 0.81 or greater for disability and 0.74 or greater for pain. The models integrated 25 routinely available preoperative features and should be readily implementable in standard clinical settings at the point of surgical decision-making.

The discriminative performance of our models is generally similar to or better than earlier prediction models for disability and pain improvement following lumbar discectomy.^10,11,14^ Staartjes et al^11^ developed a deep learning–based ODI prediction model, with a *C* statistic of 0.84. However, they only included 422 patients from a single-center registry and did not assess calibration. Similarly, Halicka et al^14^ developed disability and pain prediction models, including both patients with disc herniation and spinal stenosis. Although the models demonstrated good calibration in temporal validation data, the discriminatory ability was acceptable at best (*C* statistics from 0.62 to 0.72). In contrast, disability and pain prediction models with good discrimination (*C* statistics from 0.79 to 0.83) have been developed using data from the Danish national registry for spine surgery (DaneSpine).^10^ DaneSpine and NorSpine are very similar in terms of patient inclusion and data collection processes,^7^ but the present study is an important extension of this previous work. We used data from the whole NORspine registry (40 centers and 22 707 patients), compared with only patients from a single center of the DaneSpine (n = 1968 patients). We also used internal-external cross-validation to provide insights into heterogeneity and evaluate the generalizability of the models.

In the pursuit to develop the best prediction models, 7 machine learning algorithms were trained and tested. The algorithms showed similar discriminatory ability; however, random forest and extra trees underperformed in terms of calibration with intercepts far below 0 and slopes above 1 across all models (eTable 3 in Supplement 1). Overall, these findings are consistent with a study exploring the added value of machine learning algorithms to regression models for prognostication in traumatic brain injury.^39^ A 2019 systematic review also found no evidence of superior performance of machine learning over logistic regression in studies with relatively small sample sizes (median n = 1250).^40^ While machine learning is known to be data hungry and thrive with high-dimensional data,^41^ we did not find incremental value of our machine learning models compared with logistic regression despite the larger sample size and 25 predictor variables included. However, we emphasize that multiple models should be explored and compared when developing prediction models.

### Limitations

Our study has limitations. It was based on a large nationwide spine register using input data that align well with data available in spine registries worldwide, providing unique external validation opportunities; however, there are probably important features that we could not include due to availability, and the incremental predictive value of other predictors should be explored in external validation studies with model updating. Furthermore, the detail of some data types is also suboptimal in the NORspine registry (eg, previous medication, health care use, and work status). Including data from electronic health registries may improve predictive performance. Enrichment of our models with these data types is a subject for further work.

There are other limitations to our study. Although missingness of predictive features was low, the rate of missing outcome data was high. However, we accounted for missing data using a multiple imputation procedure, and complete-case analysis showed consistent results. Analyses from the NORspine registry also indicate that loss to follow-up does not bias conclusions about treatment effects, with no major differences in patient-reported outcomes between nonrespondents and respondents.^42,43^

Furthermore, a single agreed-on cutoff for defining benefit following lumbar discectomy is yet to be established. We chose estimates of treatment success as the outcome, calculated using anchor-based predictive modeling.^24,25^ Similar cutoffs for substantial benefit have recently been established in the Canadian Spine registry.^44^ In addition, our study sample only consisted of patients undergoing surgery (specialist health care), and their potential outcomes following nonsurgical treatment remain unknown. We argue that patients at high risk of not achieving a substantial benefit from surgery should be recommended other treatment pathways, but an impact study is needed to examine potential outcomes following nonsurgical treatment. We also acknowledge that important questions remain regarding the optimal timing of surgery,^45^ which we were not able to shed light on within our study design.

## Conclusion

We developed and validated machine learning models with high to moderate discriminative performance for predicting success or nonsuccess in disability and pain 12 months after lumbar disc herniation surgery. The models were based on routinely available preoperative predictors, making them readily amenable to further external validation in other spine registries and potentially implementable in electronic medical records systems to inform about individual prognosis and aid in surgical decision-making.

## Notes

Supplement 1. **eTable 1.** Description of Predictors **eTable 2.** Hyperparameters of Machine Learning Models **eTable 3.** Average Performance Evaluation of Machine Learning Models **eTable 4.** Predictive Values Across Validation Cohorts and Overall for Oswestry Disability Index, Numeric Rating Scale Back Pain, and Numeric Rating Scale Leg Pain **eFigure 1.** Internal-External Cross-Validation in Five Validation Cohorts and the Overall Estimation Across Validation Cohorts for (A) Numeric Rating Scale Back Pain and (B) Numeric Rating Scale Leg Pain **eFigure 2.** Assessment of Calibration in Validation Cohorts for (A) Oswestry Disability Index, (B) Numeric Rating Scale Back Pain, and (C) Numeric Rating Scale Leg Pain **eFigure 3.** Decision Curve Analysis During Internal-External Cross-Validation for (A) Oswestry Disability Index, (B) Numeric Rating Scale Back Pain, and (C) Numeric Rating Scale Leg Pain **eFigure 4.** SHAP Summary Plot of Variable Importance for Numeric Rating Scale Back Pain **eFigure 5.** SHAP Summary Plot of Variable Importance for Numeric Rating Scale Leg Pain **eFigure 6.** Internal-External Cross-Validation in Five Validation Cohorts and the Overall Estimation Across Validation Cohorts Including Only Surgical Cases With Complete Data for (A) Oswestry Disability Index, (B) Numeric Rating Scale Back Pain, and (C) Numeric Rating Scale Leg Pain

Supplement 2.
Data Sharing Statement
