# Supplementary material for: Machine Learning Models for Predicting Disability and Pain Following Lumbar Disc Herniation Surgery
Source: JAMA Netw Open. 2024 Feb 7;7(2):e2355024. doi: 10.1001/jamanetworkopen.2023.55024 (PMC10851101; doi:10.1001/jamanetworkopen.2023.55024)
Supplement: Supplement 1. — eTable 1. Description of Predictors eTable 2. Hyperparameters of Machine Learning Models eTable 3. Average Performance Evaluation of Machine Learning Models eTable 4. Predictive Values Across Validation Cohorts and Overall for Oswestry Disability Index, Numeric Rating Scale Back Pain, and Numeric Rating Scale Leg Pain eFigure 1. Internal-External Cross-Validation in Five Validation Cohorts and the Overall Estimation Across Validation Cohorts for (A) Numeric Rating Scale Back Pain and (B) Numeric Rating Scale Leg Pain eFigure 2. Assessment of Calibration in Validation Cohorts for (A) Oswestry Disability Index, (B) Numeric Rating Scale Back Pain, and (C) Numeric Rating Scale Leg Pain eFigure 3. Decision Curve Analysis During Internal-External Cross-Validation for (A) Oswestry Disability Index, (B) Numeric Rating Scale Back Pain, and (C) Numeric Rating Scale Leg Pain eFigure 4. SHAP Summary Plot of Variable Importance for Numeric Rating Scale Back Pain eFigure 5. SHAP Summary Plot of Variable Importance for Numeric Rating Scale Leg Pain eFigure 6. Internal-External Cross-Validation in Five Validation Cohorts and the Overall Estimation Across Validation Cohorts Including Only Surgical Cases With Complete Data for (A) Oswestry Disability Index, (B) Numeric Rating Scale Back Pain, and (C) Numeric Rating Scale Leg Pain [file jamanetwopen-e2355024-s001.pdf]

Supplementary Online Content

Berg B, Gorosito MA, Fjeld O, et al. Development and validation of machine learning models for predicting disability and pain following lumbar disc herniation surgery. *JAMA Netw Open*. 2024;7(2):e2355024. doi:10.1001/jamanetworkopen.2023.55024

eTable 1. Description of Predictors

eTable 2. Hyperparameters of Machine Learning Models

eTable 3. Average Performance Evaluation of Machine Learning Models

eTable 4. Predictive Values Across Validation Cohorts and Overall for Oswestry Disability Index, Numeric Rating Scale Back Pain, and Numeric Rating Scale Leg Pain

eFigure 1. Internal-External Cross-Validation in Five Validation Cohorts and the Overall Estimation Across Validation Cohorts for (A) Numeric Rating Scale Back Pain and (B) Numeric Rating Scale Leg Pain

eFigure 2. Assessment of Calibration in Validation Cohorts for (A) Oswestry Disability Index, (B) Numeric Rating Scale Back Pain, and (C) Numeric Rating Scale Leg Pain

eFigure 3. Decision Curve Analysis During Internal-External Cross-Validation for (A) Oswestry Disability Index, (B) Numeric Rating Scale Back Pain, and (C) Numeric Rating Scale Leg Pain

eFigure 4. SHAP Summary Plot of Variable Importance for Numeric Rating Scale Back Pain

eFigure 5. SHAP Summary Plot of Variable Importance for Numeric Rating Scale Leg Pain

eFigure 6. Internal-External Cross-Validation in Five Validation Cohorts and the Overall Estimation Across Validation Cohorts Including Only Surgical Cases With Complete Data for (A) Oswestry Disability Index, (B) Numeric Rating Scale Back Pain, and (C) Numeric Rating Scale Leg Pain

This supplementary material has been provided by the authors to give readers additional information about their work.

© 2024 Berg B et al. *JAMA Network Open.*

eTable 1. Description of predictors

Predictor	Description	Categorization
Sex	Male or female	Binary
Age	Estimated in years, using date of birth from the personal identifier and date of surgical admission	Continuous
Body mass index	Based on self-reported height and weight, in kg/m^2	Continuous
Native language	Native or non-native speaker	Binary
Marital status	Single or partner/married	Binary
Smoking	Non-smoker or current smoker	Binary
Education	Highest education level at the time of surgical admission, categorized as lower secondary school, upper secondary school, college/University 1-3 years, college/University >3 years	Categorical
Work status	At the time of surgical admission, categorized as working or student, age retirement, sick leave, disability pension/work assessment allowance	Categorical
Litigation issue	Pending medical claim/litigation against the Norwegian public welfare agency found concerning disability pension or against insurance companies/the public Norwegian System of Compensation to Patients	Binary
Anxiety/Depression	Self-reported anxiety or depression, based on EQ-5D 5th item: moderate to severe (3L) or extreme (5L)	Binary
Comorbidities	Recorded by the surgeon, from a list: rheumatoid arthritis, osteoarthritis, depression/anxiety, generalized pain syndrome, chronic neurological disease, cerebrovascular disease, cardiovascular disease, chronic pulmonary disease, cancer, osteoporosis, hypertension, diabetes mellitus, other endocrine disorders. Categorized as none, one, two, three or more	Categorical
ASA grade	American Society of Anesthesiologists grade recorded by the surgeon, categorized as 0-2 or 3-5	Binary
ODI	Oswestry Disability Index score (range 0 to 100)	Continuous
NRS back pain	Numeric Rating Scale for back pain (range 0 to 100)	Continuous
NRS leg pain	Numeric Rating Scale for leg pain (range 0 to 100)	Continuous
EQ-5D	Health-related quality of life (5L values). For patients responding to the 3L version, reverse crosswalk values were computed using the EQ-5D-5L Delvin value set (range -0.825 to 1)	Continuous
Health status	Self-reported health status using Visual Analogue Scale from EQ-5D (range 0 to 100)	Continuous
Back pain duration	Self-reported duration of back pain, categorized as less than 3 months, 3 to 12 months, 12 to 24 months, more than 24 months.	Categorical
Leg pain duration	Self-reported duration of leg pain, categorized as less than 3 months, 3 to 12 months, 12 to 24 months, more than 24 months.	Categorical
Analgesics use	Self-reported frequency due to back pain, categorized as monthly, weekly, daily.	Categorical
Paresis grade	Based on manual muscle testing performed by the surgeon using a scale from 0 to 5, categorized as normal (grade 5), mild (grade 4), severe (grade 0-3)	Categorical
Previous surgery	Number of previous surgeries recorded by the surgeon, categorized as none, one, two or more	Categorical
Microdiscectomy	Type of surgery; microdiscectomy or open discectomy	Binary
Surgical levels	Number of surgical levels operated on, categorized as one level, two or more levels	Binary
Emergency surgery	Elective or emergency surgery	Binary

ASA=American Society of Anesthesiologists grade; ODI=Oswestry Disability Index; NRS=Numeric Rating Scale.

© 2024 Berg B et al. *JAMA Network Open.*

eTable 2. Hyperparameters of machine learning models

Model	Hyperparameter	Searched value or category	Chosen value or category		
			ODI	NRS back	NRS leg
RF	Max depth	6, 7, 8, 9	9	9	9
	Max features	6, 7, 8, 9	9	9	8
	Min samples split	5, 6	5	6	5
	N of estimators	500, 1000, 10000	1000	10000	500
LR	C	0.1, 1, 10, 100	100	100	100
	Solver	Saga, liblinear, lbfgs, sag	Saga	Saga	Saga
LDA	Solver	Svd, lsqr, eigen	Svd	Svd	Svd
MLP	Hidden layer	4, 8, 16	8	8	8
	Activation	Logistic, relu, tanh	Logistic	Logistic	Logistic
	Batch size	256, 512, 1024	1024	512	1024
GB	Learning rate	0.005, 0.01, 0.1	0.01	0.01	0.005
	Max depth	5, 6, 7, 8	5	5	5
	Max features	null, 5, 8, 10	8	8	8
	Min samples leaf	5, 10	5	10	10
	N of estimators	500, 1000, 1500	1500	1000	1500
Extra Trees	Max depth	7, 8, 9, 10	10	10	10
	N of estimators	100, 500, 1000	500	1000	500
	Min samples leaf	5, 50	5	50	5
	Max features	0.2, 0.5, 0.9	0.9	0.5	0.5
XGB	Learning rate	0.005, 0.01, 0.1	0.1	0.1	0.1
	Max depth	1, 2, 4, 8, 10	1	1	1
	N of estimators	100, 500, 1000	1000	1000	500

ODI=Oswestry Disability Index, NRS=Numeric Rating Scale; RF=Random forest; N=Number; LR=Logistic regression; LDA=Linear discriminatory analysis; MLP=Multi-layer perceptron; GB=Gradient boosting; XGB=XGBoost

© 2024 Berg B et al. *JAMA Network Open.*

eTable 3. Average performance evaluation of machine learning models[a]

		Average performance, mean (95% CI)		
**Outcome**	**Model**	**C-statistic**	**Calibration intercept**	**Calibration slope**
ODI	RF	0.81 (0.79, 0.83)	-0.23 (-0.34, -0.11)	1.26 (1.18, 1.35)
	LR	0.82 (0.80, 0.84)	-0.03 (-0.11, 0.05)	0.98 (0.92, 1.04)
	LDA	0.82 (0.80, 0.84)	0.02 (-0.06, 0.10)	1.00 (0.93, 1.06)
	MLP	0.82 (0.80, 0.84)	0.01 (-0.07, 0.10)	0.99 (0.92, 1.06)
	GB	0.82 (0.80, 0.84)	-0.04 (-0.14, 0.05)	0.96 (0.90, 1.02)
	Extra Trees	0.81 (0.80, 0.83)	-0.07 (-0.16, 0.02)	1.09 (1.02, 1.16)
	XGB	0.82 (0.81, 0.84)	0.01 (-0.07, 0.10)	0.99 (0.93, 1.06)
NRS back pain	RF	0.76 (0.74, 0.78)	-0.26 (-0.34, -0.17)	1.34 (1.24, 1.44)
	LR	0.77 (0.75, 0.79)	0.02 (-0.05, 0.10)	0.98 (0.92, 1.04)
	LDA	0.77 (0.75, 0.79)	0.04 (-0.03, 0.11)	0.95 (0.89, 1.01)
	MLP	0.77 (0.74, 0.79)	0.01 (-0.06, 0.09)	0.99 (0.93, 1.06)
	GB	0.77 (0.74, 0.79)	0.01 (-0.07, 0.09)	1.00 (0.93, 1.07)
	Extra Trees	0.76 (0.74, 0.79)	-0.18 (-0.26, -0.10)	1.23 (1.15, 1.32)
	XGB	0.77 (0.74, 0.79)	0.00 (-0.07, 0.08)	1.01 (0.94, 1.07)
NRS leg pain	RF	0.74 (0.73, 0.76)	-0.23 (-0.36, -0.10)	1.33 (1.23, 1.44)
	LR	0.75 (0.74, 0.76)	0.03 (-0.07, 0.13)	0.96 (0.88, 1.04)
	LDA	0.75 (0.74, 0.76)	0.06 (-0.04, 0.16)	0.92 (0.85, 1.00)
	MLP	0.75 (0.74, 0.76)	0.02 (-0.09, 0.13)	0.97 (0.89, 1.06)
	GB	0.75 (0.74, 0.76)	-0.01 (-0.13, 0.11)	1.02 (0.93, 1.12)
	Extra Trees	0.74 (0.73, 0.75)	-0.07 (-0.18, 0.04)	1.11 (1.03, 1.18)
	XGB	0.75 (0.74, 0.76)	-0.01 (-0.14, 0.11)	1.02 (0.92, 1.12)

ODI=Oswestry Disability Index; NRS=Numeric Rating Scale; RF=Random forest; LR=Logistic regression; LDA=Linear discriminatory analysis; MLP=Multi-layer perceptron; GB=Gradient boosting; XGB=XGBoost
[a]Average test performance across all cluster combined using random-effects meta-analysis

© 2024 Berg B et al. *JAMA Network Open.*

eTable 4. Predictive values across validation cohorts and overall for Oswestry Disability Index, Numeric Rating Scale back pain, and Numeric Rating Scale leg pain

	**ODI**		**NRS back pain**		**NRS leg pain**	
	PPV (95% CI)	NPV (95% CI)	PPV (95% CI)	NPV (95% CI)	PPV (95% CI)	NPV (95% CI)
South. East.	0.81 (0.80, 0.83)	0.63 (0.61, 0.65)	0.83 (0.81, 0.84)	0.49 (0.47, 0.52)	0.80 (0.78, 0.82)	0.54 (0.52, 0.56)
Western	0.84 (0.82, 0.86)	0.61 (0.59, 0.64)	0.84 (0.82, 0.86)	0.49 (0.46, 0.52)	0.81 (0.78, 0.82)	0.54 (0.51, 0.56)
Central	0.87 (0.85, 0.89)	0.58 (0.55, 0.62)	0.86 (0.84, 0.88)	0.47 (0.44, 0.49)	0.83 (0.81, 0.85)	0.52 (0.49, 0.54)
Northern	0.87 (0.85, 0.89)	0.58 (0.53, 0.62)	0.88 (0.86, 0.90)	0.50 (0.46, 0.54)	0.81 (0.79, 0.84)	0.50 (0.46, 0.54)
Private	0.88 (0.87, 0.90)	0.51 (0.48, 0.53)	0.88 (0.87, 0.90)	0.42 (0.39, 0.45)	0.87 (0.86, 0.89)	0.40 (0.37, 0.43)
Overall	0.86 (0.82, 0.89)	0.58 (0.52, 0.64)	0.86 (0.83, 0.89)	0.47 (0.43, 0.51)	0.83 (0.79, 0.86)	0.50 (0.43, 0.57)

ODI=Oswestry Disability Index, NRS=Numeric Rating Scale, PPV=Positive predictive value, NPV=Negative predictive value; South. East=Southern and Eastern

© 2024 Berg B et al. *JAMA Network Open.*

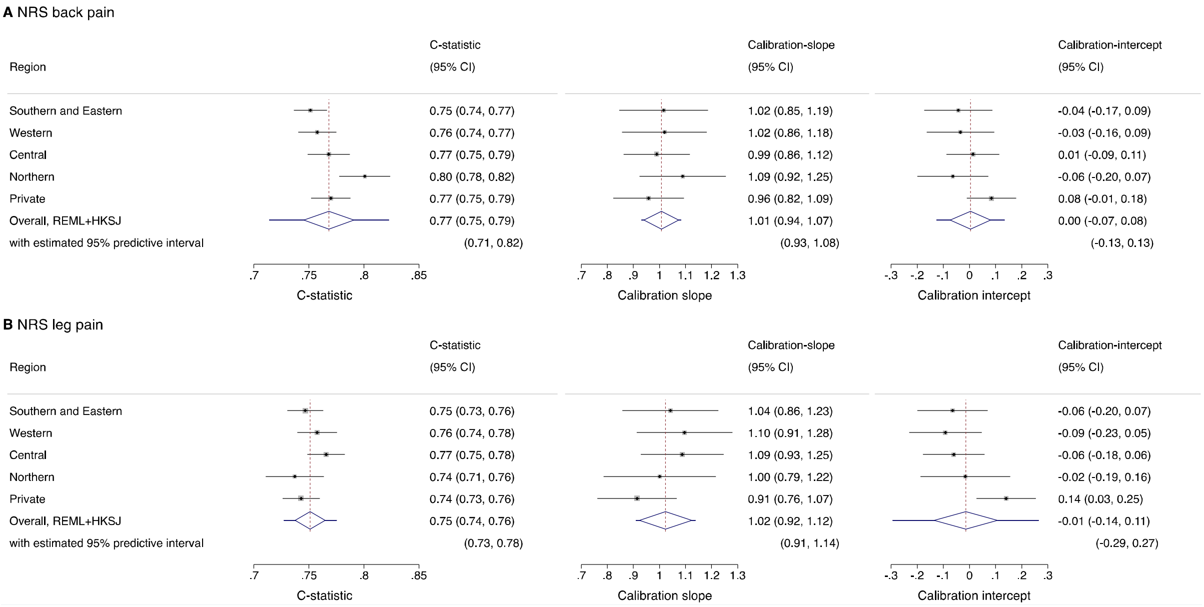

eFigure 1. Internal-external cross-validation in five validation cohorts and the overall estimation across validation cohorts for (A) Numeric Rating Scale back pain and (B) Numeric Rating Scale leg pain.

© 2024 Berg B et al. *JAMA Network Open.*

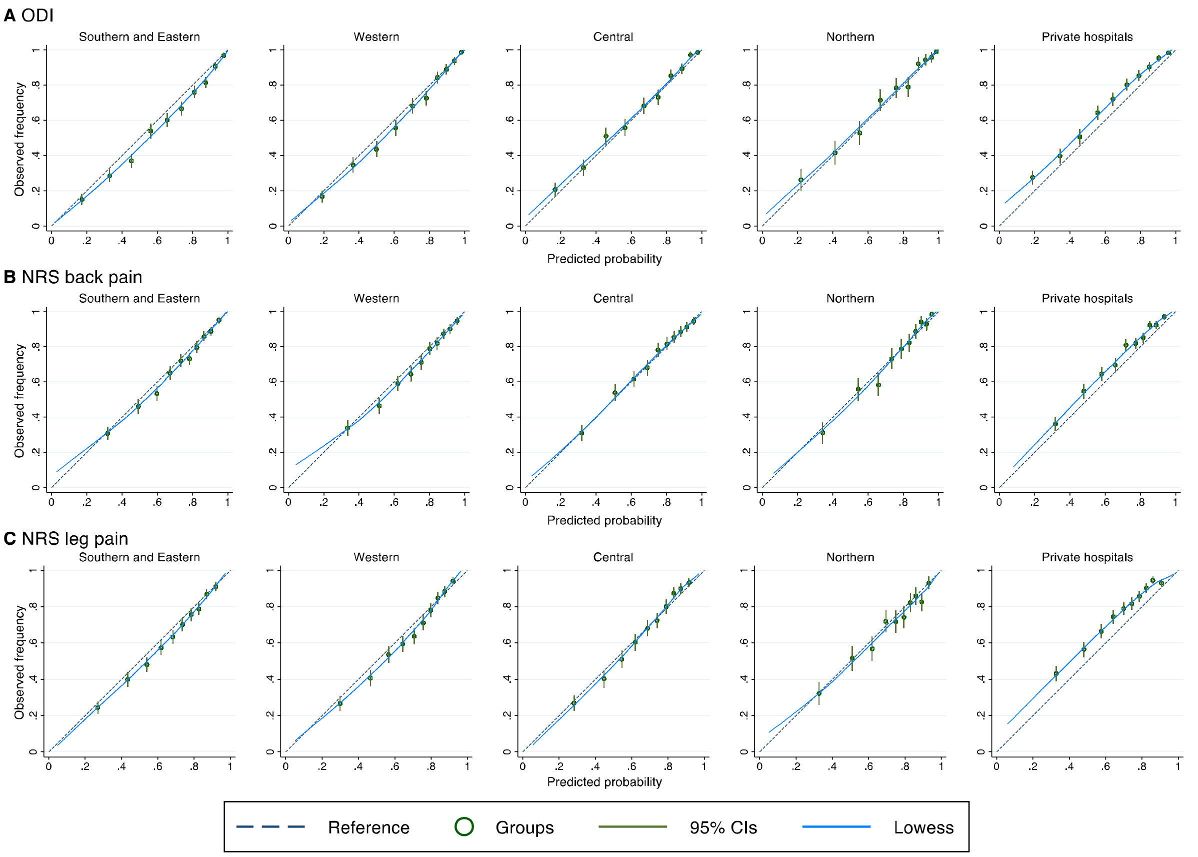

eFigure 2. Assessment of calibration in validation cohorts for (A) Oswestry Disability Index, (B) Numeric Rating Scale back pain, and (C) Numeric Rating Scale leg pain

© 2024 Berg B et al. *JAMA Network Open.*

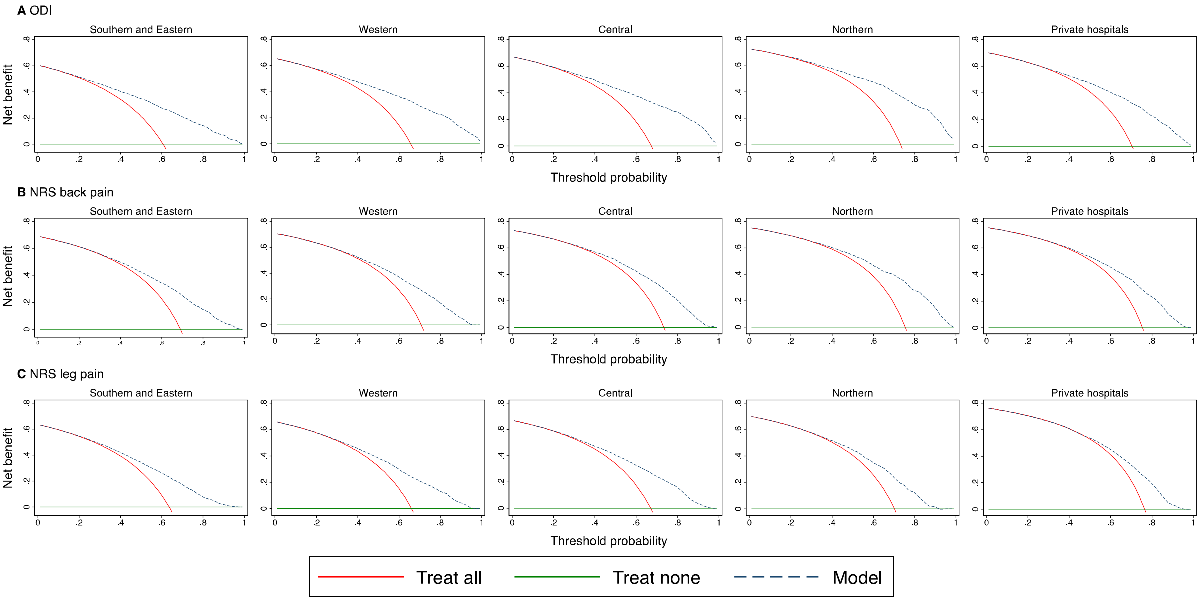

eFigure 3. Decision Curve Analysis during internal-external cross-validation for (A) Oswestry Disability Index, (B) Numeric Rating Scale back pain, and (C) Numeric Rating Scale leg pain

© 2024 Berg B et al. *JAMA Network Open.*

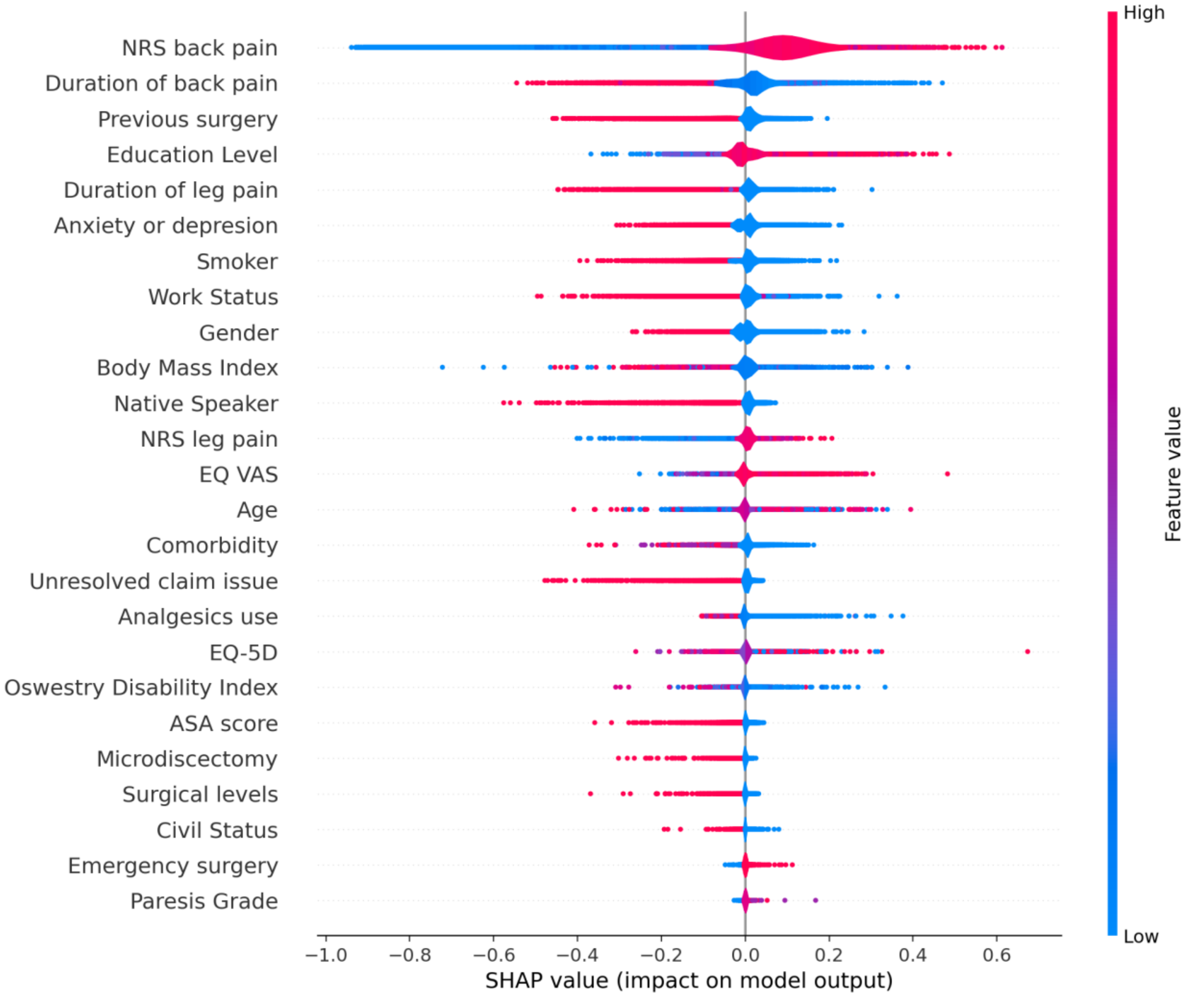

eFigure 4. SHAP summary plot of variable importance for Numeric Rating Scale back pain. Predictive features are arranged along the y-axis based on their importance. Each dot represents one prediction result, with the colors indicating high (red) to low (blue) feature values. SHAP values on the x-axis indicate the distribution of the prediction among the features; a positive value contributes to treatment success, while a negative value contributes to non-success.

© 2024 Berg B et al. *JAMA Network Open.*

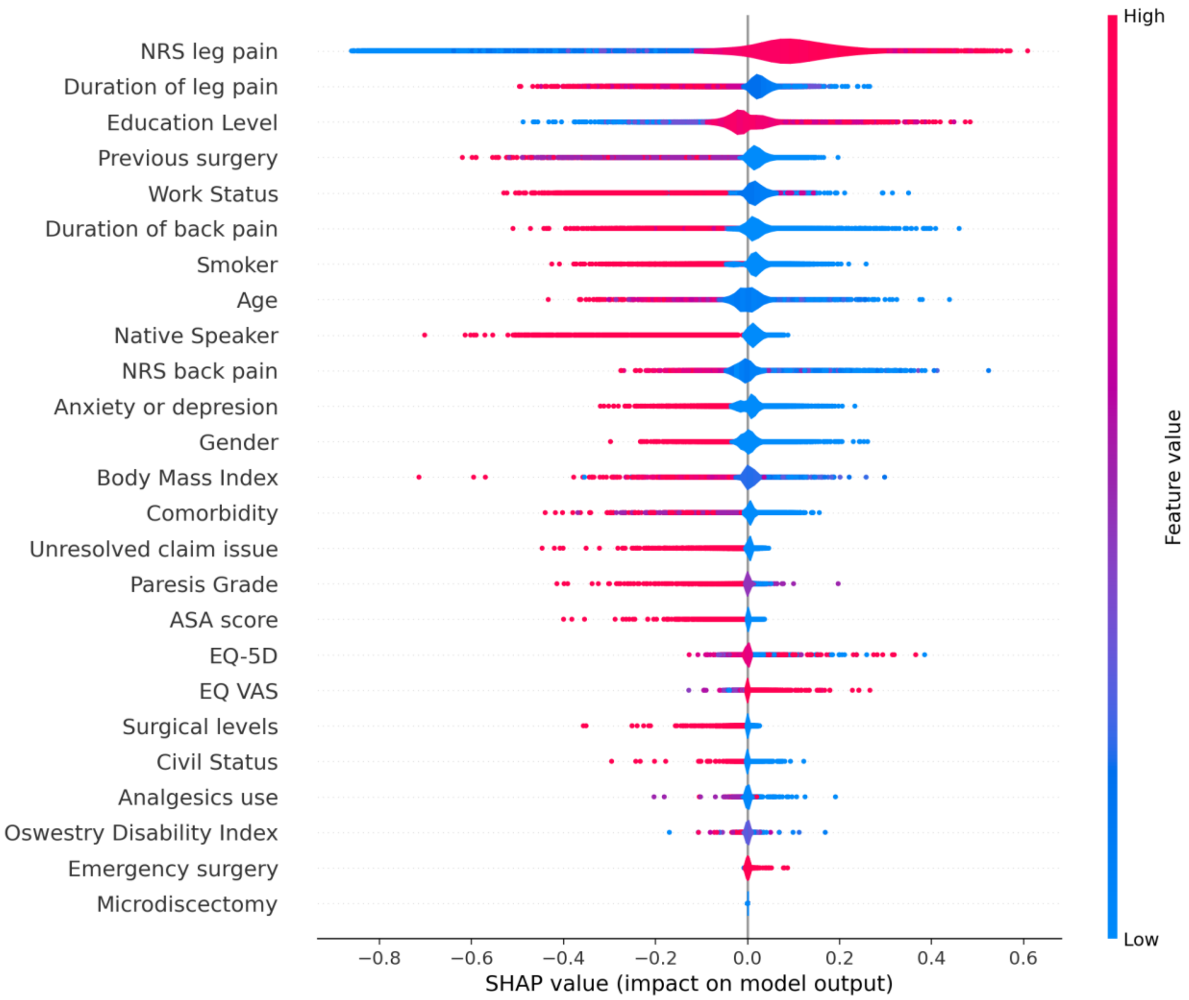

eFigure 5. SHAP summary plot of variable importance for Numeric Rating Scale leg pain. Predictive features are arranged along the y-axis based on their importance. Each dot represents one prediction result, with the colors indicating high (red) to low (blue) feature values. SHAP values on the x-axis indicate the distribution of the prediction among the features; a positive value contributes to treatment success, while a negative value contributes to non-success.

© 2024 Berg B et al. *JAMA Network Open.*

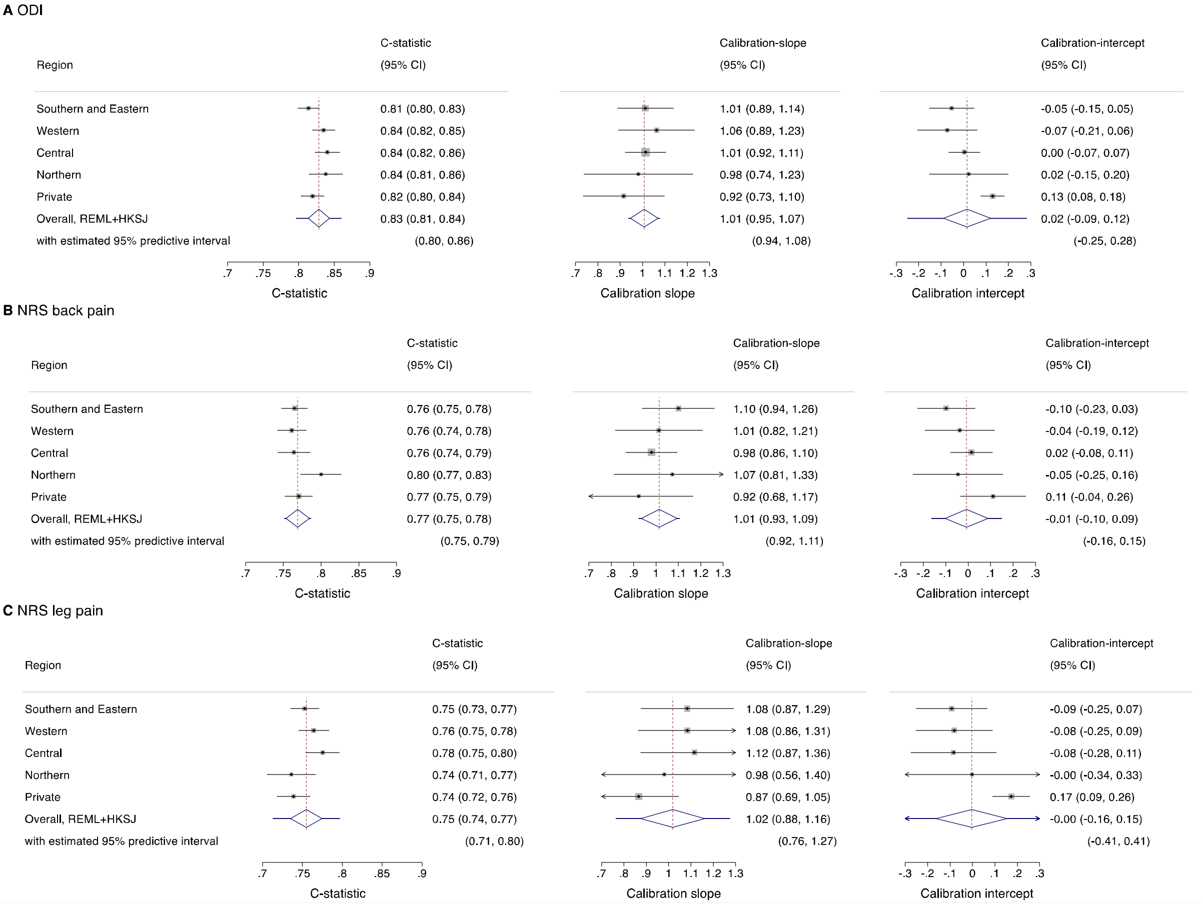

eFigure 6. Internal-external cross-validation in five validation cohorts and the overall estimation across validation cohorts including only surgical cases with complete data for (A) Oswestry Disability Index, (B) Numeric Rating Scale back pain, and (C) Numeric Rating Scale leg pain.

© 2024 Berg B et al. *JAMA Network Open.*
