# Supplementary material for: Machine Learning Models for Predicting Disability and Pain Following Lumbar Disc Herniation Surgery
Source: JAMA Netw Open. 2024 Feb 7;7(2):e2355024. doi: 10.1001/jamanetworkopen.2023.55024 (PMC10851101; doi:10.1001/jamanetworkopen.2023.55024)
Supplement: Supplement 2. — Data Sharing Statement [file jamanetwopen-e2355024-s002.pdf]

Data Sharing Statement

Berg. Machine Learning Models for Predicting Disability and Pain Following Lumbar Disc Herniation Surgery. *JAMA Netw Open*. Published February 07, 2024.
doi:10.1001/jamanetworkopen.2023.55024

Data
Data available: No

Additional Information
Explanation for why data not available: The analytic code is available from the authors upon request.
